# Treatment of Metastatic Colorectal Cancer Patients ≥75 Years Old in Clinical Practice: A Multicenter Analysis

**DOI:** 10.1371/journal.pone.0157751

**Published:** 2016-07-21

**Authors:** Roberta Grande, Clara Natoli, Fabrizio Ciancola, Donatello Gemma, Arianna Pellegrino, Ida Pavese, Carlo Garufi, Luigi Di Lauro, Domenico Corsi, Diego Signorelli, Isabella Sperduti, Giada Cortese, Emanuela Risi, Federica Morano, Domenico Sergi, Carlo Signorelli, Enzo Maria Ruggeri, Germano Zampa, Marco Russano, Teresa Gamucci

**Affiliations:** 1 Medical Oncology Unit, ASL Frosinone, Frosinone, Italy; 2 Medical Oncology Unit, G. D'Annunzio University-Chieti, Chieti, Italy; 3 Medical Oncology Unit, La Sapienza University-Rome, Rome, Italy; 4 Medical Oncology Unit, San Pietro Fatebenefratelli Hospital, Rome, Italy; 5 Medical Oncology Unit, Regina Elena Cancer Institute, Rome, Italy; 6 Medical Oncology Unit, S. Giovanni Calibita Fatebenefratelli Hospital, Isola Tiberina-Rome, Rome, Italy; 7 Department of Bio-Statistics, Regina Elena National Cancer Institute, Rome, Italy; 8 Medical Oncology Unit, Belcolle Hospital, Viterbo, Italy; 9 Medical Oncology Unit ASL RM/A, Rome, Italy; 10 Medical Oncology Unit, Campus Biomedico University, Rome, Italy; University of Algarve, PORTUGAL

## Abstract

**Background:**

Colorectal cancer patients have a median age of incidence >65years although they are largely under-represented in phase-III trials. This large population contains patients unfit for treatment, those suitable for monotherapy or for doublets and the impact of chemotherapy outside clinical trial is unclear. The aim of the study was to retrospectively analyse Overall Survival(OS) of elderly metastatic colorectal cancer(mCRC) patients treated with chemotherapy in daily practice.

**Methods:**

Kaplan-Meir method was used for OS, the log-rank or Tarone-Ware test for differences between subgroups, Cox’s proportional hazard model to assess the impact of known prognostic factors and treatment.

**Results:**

751 patients with mCRC observed between January 2000 and January 2013 were collected. Median age was 79 year(75–93); Male/Female 61/39%, ECOG-PS 0-1/2 85/15%; colon/rectum 74/26%; multiple metastatic sites 34%, only liver metastasis in 41% of patients. KRAS status was studied in 35% of patients: 44% of them showed gene mutation. 20.5% of patients did not received any kind of treatment including surgery. Comorbidities observed: cardiovascular 34%, diabetes 14%, hypertension 50%. Primary tumor was resected in 80.6%; surgery of liver metastasis was done in 19% of patients (2.3% of patients >80years). 78% of patients underwent chemotherapy. Median follow up was 12 months(range 1–124). Median OS was 17 months (CI 95%15–19);median OS in no-treated patients was 5 months (4–6); mOS of patients with at least one treatment was 20 months (18–22). In KRAS mutated group median OS was 19months (15–23) while in KRAS wild type patients median OS was 25 months (20–30). At multivariate analysis sex(Female), age(<80y), performance status(0–1), chemotherapy, Surgery of metastasis, Surgery of primary tumor and Site of metastasis(liver) were prognostic factors for OS.

**Conclusion:**

The results of our study show that in clinical practice treatment has a positive impact on OS of elderly patients, confirmed at multivariate analysis, included patients with age >80 years old or with a poor performance status (respectively p<0.0001 and p<0.0001). KRAS analysis deserve further evaluation.

## Introduction

Colorectal cancer (CRC) is one of the most common cancers and the number of new cases diagnosed each year is expected to increase due to the higher life expectancy.

The results achieved through the advances in surgery and chemotherapy prolonged time to disease progression and survival in patients with metastatic disease [1].

Colorectal cancer patients have a median age of incidence >65 years and the percentage of patients >75 and >85 years old will increase disproportionately [2–4].

This group of patients is significantly underrepresented in clinical trials [5–8] because of the careful selection (age restriction in study enrolment, good performance status and comorbidity). Moreover, the definition of ‘‘elderly” is not so clear and it could often include patients aged from >65 years to patients aged >75 years. [9–12]

Therefore the selection of optimal treatment is very complex and the result is the lack of specific data about this population, in particular for patients over 80.

There is evidence that the essential principles of treating advanced cancer in the elderly are the same as in younger patients. Elderly patients, who have age-related organ function decline and comorbidity, require special attention to the risks of toxicity of treatment and quality of life [12].

Age alone should not be used to deny potentially beneficial treatment to any patient with cancer. Older patients, as a population, present considerable heterogeneity with regard to co-morbidity and functional status. In fact this large population includes patients unfit for treatment, those suitable for monotherapy or for doublets and the impact of chemotherapy outside clinical trial is unclear; furthermore fit older patients could be enrolled in clinical trials. Until 2013 the number of elderly patients was too small in clinical trials and no clear data was available about the impact of treatment on their survival.

Available data about elderly population showed that antitumor efficacy of chemotherapy is similar to that in younger patients, although there is a higher rate of hematologic toxicity. Based upon these results, a 5-FU-based chemotherapy regimens in combination with oxaliplatin or irinotecan should be a feasible option [13]. Most elderly patients could tolerate the addition of bevacizumab to these regimens for first-line therapy, although potential thromboembolic complications are deterring factor that influence its use.

In 2013 AVEX trial showed first data about toxicities and efficacy of first line chemotherapy regimen with the association of capecitabine and bevacizumab in elderly patients. Results from this trial showed that the addition of bevacizumab to capecitabine significantly improved progression-free survival in elderly patients with metastatic colorectal cancer with a safety profile [14].

Recently Rouyer M. et all added new data about the safe and beneficial effect of first line treatment with bevacizumab plus Folfiri in elderly ptients with mCRC [15].

Trials available regarding the use of cetuximab in elderly patients showed activity of the drug in this category of patients but no phase III trials exclusive to the elderly are available or the age sample limit was 65 years of age [16–17].

In Crystal and Opus studies [18–19] the addition of cetuximab improved the efficacy of chemotherapy alone without increasing toxicities but the number of elderly observed is too small.

In the “panitumumab-study” the median age was 62 years and patients above 80 years were also included but no detailed data were shown about this group [20].

Based on these data, it is necessary to evaluate how elderly patients were treated in clinical practice and what is the advantage obtained from treatment.

The aim of this study was to retrospectively evaluate OS of elderly metastatic colorectal cancer patients treated with chemotherapy in daily practice.

## Materials and Methods

From January 2000 to January 2013, 751 patients > 75 years old with metastatic colorectal cancer were retrospectively collected and analyse from ten Italian centres. The patients enrolled received any kind of treatment before (surgery alone, chemotherapy alone or both) or only the best supportive care defined as the best palliative treatment for investigators to maximize the quality of life, excluding antineoplastic agents.

From November 2008 we had the opportunity to detect mutation in codon 12 and 13 of KRAS gene; the analyses was performed at each individual center. Patient information was anonymized and de-identified prior to analysis. The study was approved by local institutional ethic committees (Ethic Committees “Lazio 2”).

### Statistical method

The follow-up was analyzed and reported according to Shuster et al [21]. The association between variables was tested by the Pearson Chi-Square test or Fisher exact test, when appropriate. The Hazard Ratio (HR) and the 95% confidence intervals (95% CI) was estimated for each variable using the Cox univariate model. A multivariate Cox proportional hazard model was also developed using stepwise regression (forward selection). Enter limit and remove limit will be p = 0.10 and p = 0.15 respectively. The assessment of interactions between significant investigation variables was taken into account when developing the multivariate model.

To reduce the selection biases related to a non randomized cohort, a propensity score for the likelihood of receiving chemotherapy or not, was calculated from 3 covariates: age, performance status and surgery of primary tumor. By using a 1:1 nearest neighbor matching algorithm that pairs patients with the closest propensity scores within a defined limit (calipers of width equal to 0.2), the propensity score yielded 2 well-matched cohorts of 268 patients (logistic regression estimation algorithm).

Multivariate logistic regression analysis was used to assess the impact of variables on the variable therapy.

Overall survival (OS) was calculated by the Kaplan-Meier product-limit method from the date of the diagnosis until death. Survival curves was truncated at that time-point where the recommendations according to Pocock et al [22] were satisfied. The analysis of OS was performed dividing patients into two groups: patients treated with chemotherapy (group A) and no-treated (group B) with chemotherapy. The log-rank test or Traone-Ware test were used to assess differences between subgroups. Significance was defined at the p<0.05 level. The SPSS^®^(21.0),statistical program was used for all analyses.

## Results

Patient's characteristics are listed in Table 1. Median number of patients per center was 75. We identified 441 patients <80 years old and 310 patients with age ≥80; 9.2% of patients was ≥84 years old. Cardiovascular disease was present in 34.5% of patients, diabetes in 14.5% and hypertension in 49.7%.

**Table 1:** Patient Characteristics (N = 751).

**Category**	**Subcategory**	**N° of patients (%)**
Median age (range)		79 (75–93)
	<80years	441(58.7%)
	≥80 years	310 (41.3)
Median follow up (range)		12 months (1–124)
Sex	Men	461 (61.4)
	Women	290 (38.6)
Tumor location	colon	558 (74.3)
	rectal	193(25.7)
Grading	1	15 (2.0)
	2	324(43.1)
	3	249 (33.2)
	Unknown	163 (21.7)
ECOG PS	0/1	641 (85.4)
	2	110 (14.6)
Comorbidities	Cardiovascular disease	259(34.5)
	Diabetes	109(14.5)
	Hypertension	373(49.7)
KRAS	Wild Type	148 (19.7)
	Mutant	117 (15.6)
	UnKnown	486 (64.7)
Synchronous disease		439 (58.5)
Site of metastasis	Liver only	309 (41.1)
	Lung only	77 (10.3)
	Multi-organ	258 (34.4)
	Other site	9 (1.2)
Surgery of primary tumor		605 (80.6)
Surgery of metastasis		127 (16.9)
Adjuvant treatment		143 (46)
First line Chemotherapy	Any	578 (78)
	Monotherapy	178(30.8)
	Target therapy	187(32.3)
Second line Chemotherapy		274 (47.4)
Third line Chemotherapy		119 (15.8)

Multiple site of metastatic disease was diagnosed in 34% and 41% of patients had only liver disease. Kras status was studied in 35% of patients: of them 44% showed gene mutation.

153 patients did not received any kind of treatment including surgery: 67% of them were ≥80years old.

**Fig 1:**
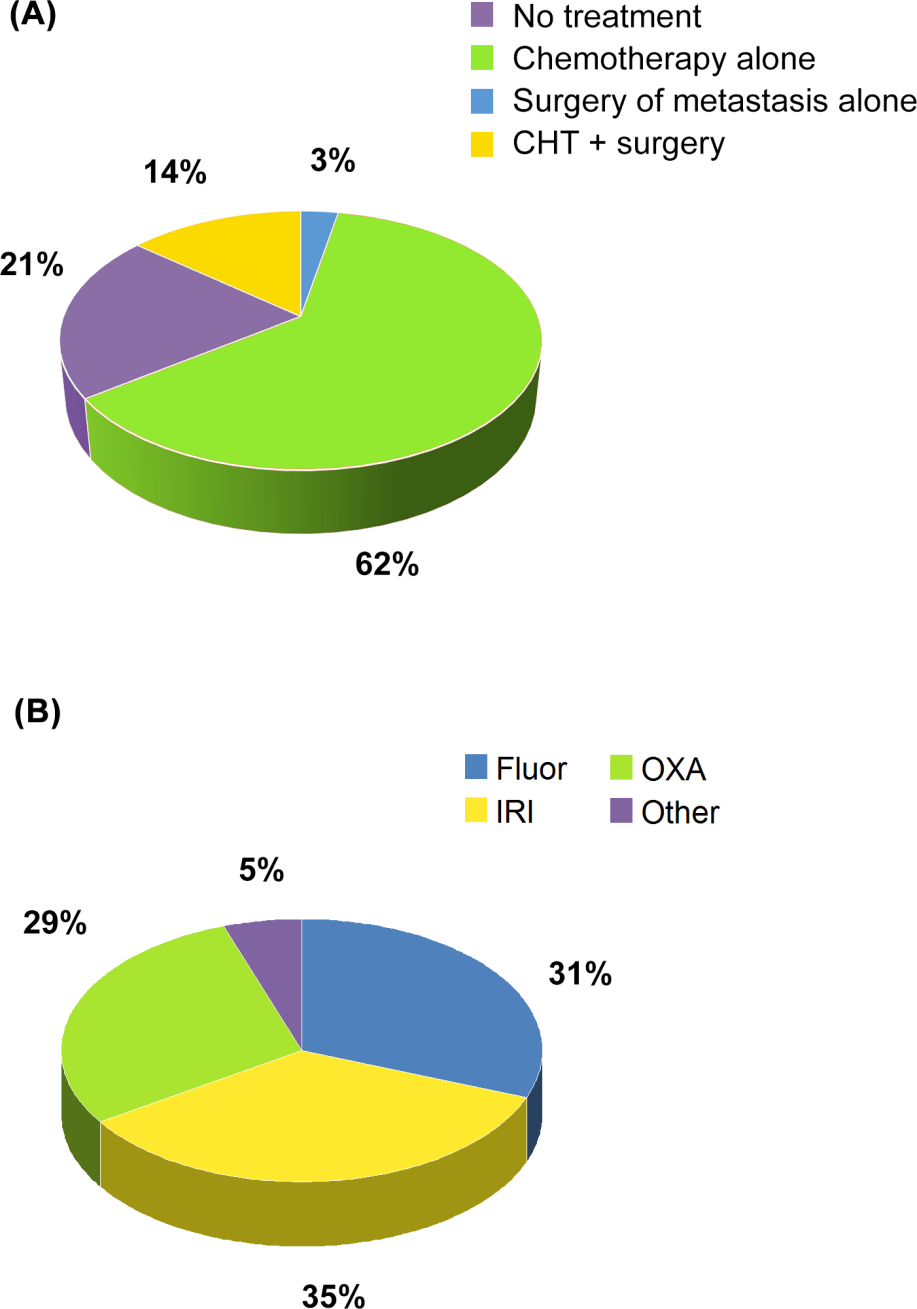
Distribution of patients by treatment (A) and kind of treatment (B).

The younger group of patients (<80 years old) had a higher treatment rate *across all treatment*. The treatment received by patients and the type of chemotherapy are summarized in Fig 1.

Primary tumor was resected in 80.6% of patients. Synchronous disease was observed in 58.5% of patients: in this subgroup, surgery of primary tumor was performed for symptoms at diagnosis (data not shown). Liver-only metastases were reported in 309 patients (41%) and metastatectomy was done in 21.1% of them (65 patients): median age of patients was 78 years (range 74–86); 20 patients (30.8%) had age ≥80 years.

Local radiotherapy was performed in 34% of rectal cancers.

578 patients (78%) received chemotherapy: of them,14% of patients underwent to surgery of metastasis. 32.3% (187 patients) received target therapies with monoclonal antibodies. Monochemotherapy was performed in 30.8% (178) of treated patients and 95 patients (53.4%) were ≥80years old.

274 patients (47.4%) underwent second line chemotherapy and only 29.9% (82 patients) received target therapies.

43.4% of patients (119 patients) who underwent second line chemotherapy reached third line of treatment and 43.7% (52 patients) of them received further treatments.

In the majority of cases, patient treatment was discontinued because of disease progression in all line of treatment (about 56%); about 12.5% of patients discontinued treatment due to toxicities or patients refusal. The toxicities observed were in line with those expected from the regimen administered.

The median follow up was 12 months (range 1–124).

**Fig 2:**
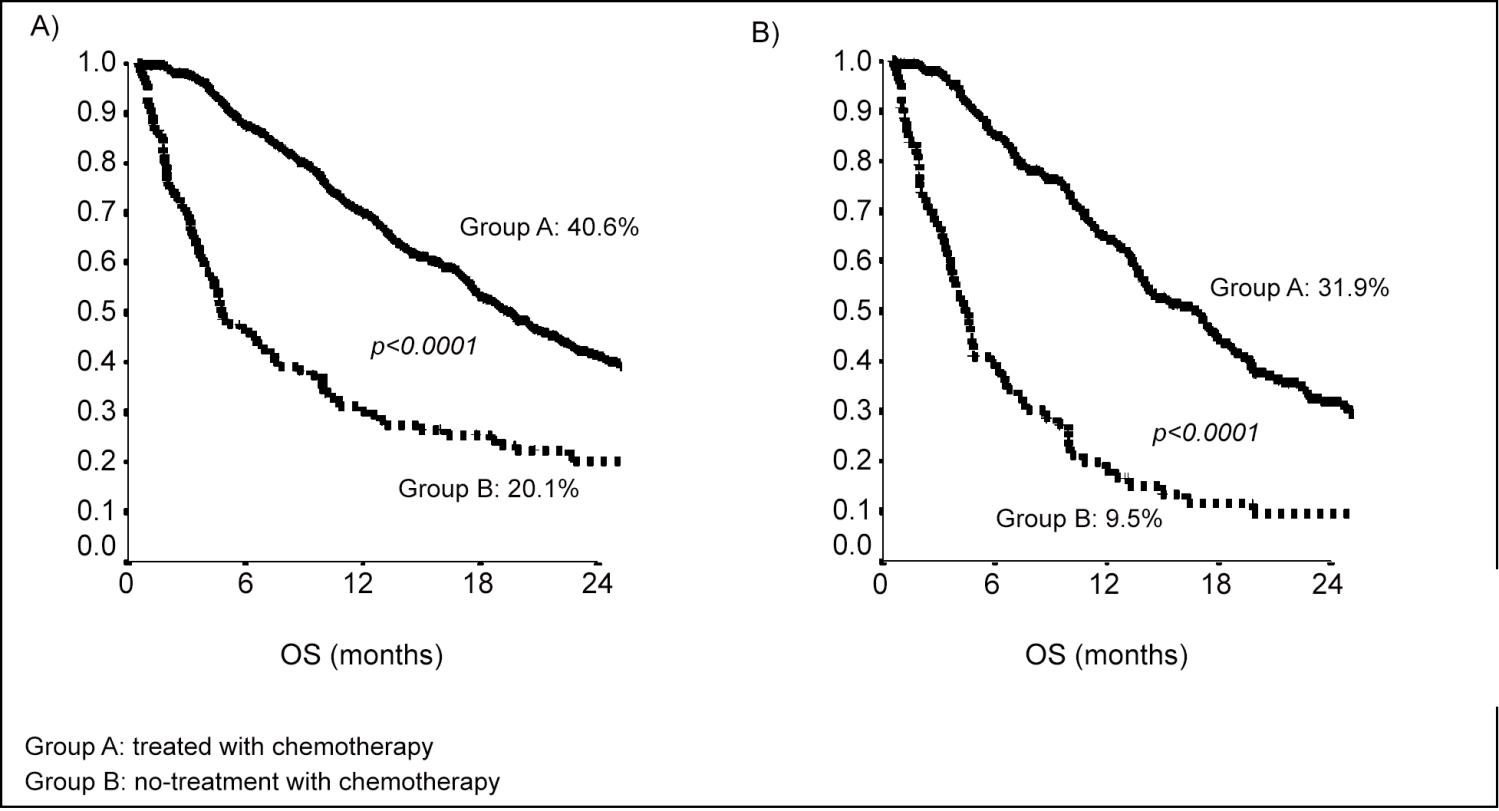
Kaplan-Meier curves of 2-years Overall Survival: patients treated with chemotherapy (A); patients ≥80 years old (B).

Median overall survival (OS) was 17 months (CI95%15–19); in group B (patients no-treated with chemotherapy) median OS was 5 months (CI95% 4–6); median OS of patients who underwent at least at one treatment of chemotherapy (group A) was 20 months (CI95% 18–22) Fig 2A.

Median OS of patients who underwent to only resection of metastasis (19 patients of group B) was 22 months. Median OS of patients treated with target therapies was 23 months (CI 95% 19–26).

The analysis of OS by age evidenced that 2-years OS in patients with <80 years was 48.1% in group A and 44% in group B (p = 0.43); 2-years OS in patients with ≥80 years was 34.8% in group A and 17.6% in group B (p<0.0001) Fig 2B.

**Fig 3:**
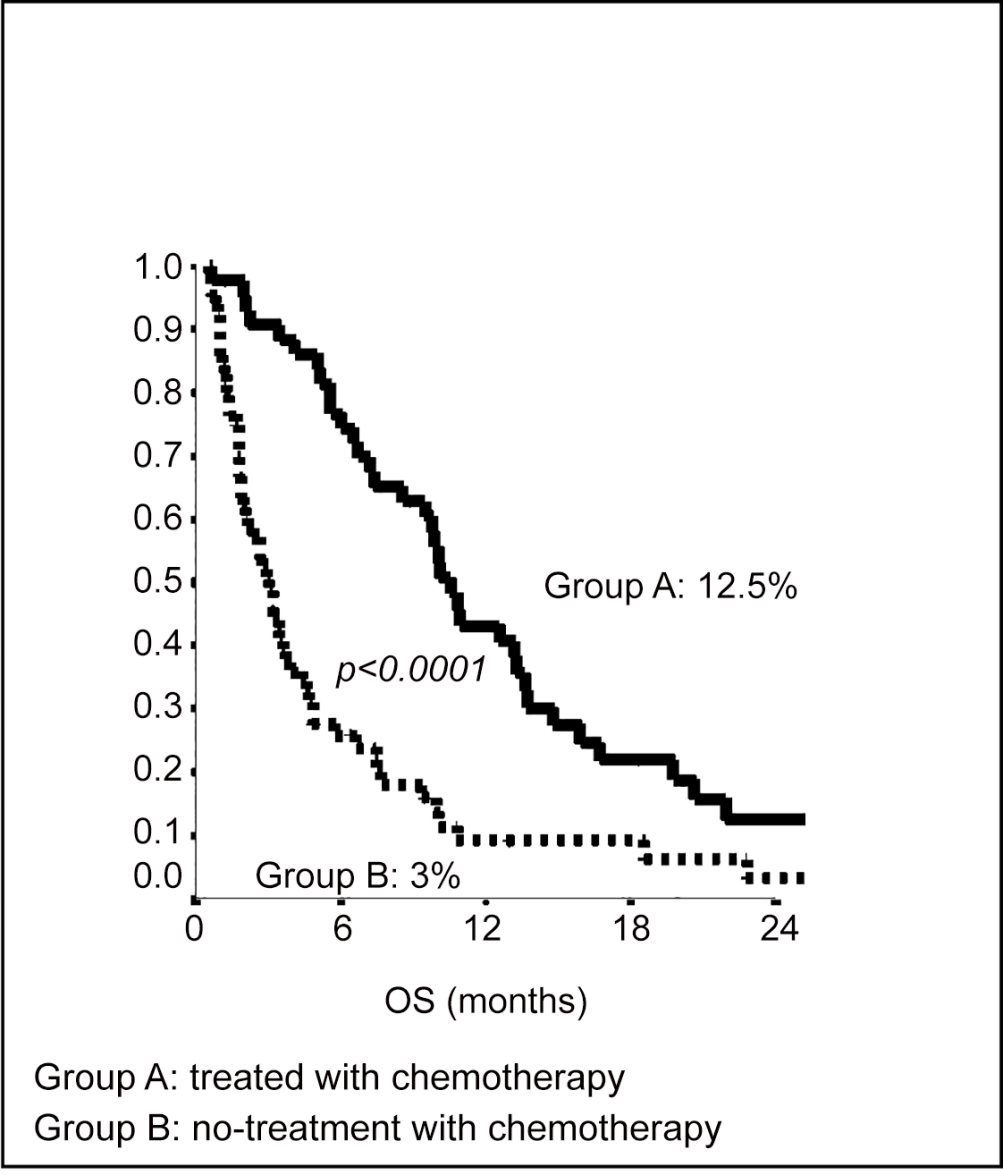
Kaplan-Meier curves of 2-years Overall Survival of patients with poor ECOG PS.

When we analyzed OS by performance status we noted that 2-years OS in patients with ECOG PS 0–1 was 46.2% in group A and 41% in group B (p = 0.21); 2-years OS in patients with ECOG PS 2 was 12.8% in group A and 8.1% in group B (p<0.0001) Fig 3.

The analysis of KRAS status revealed that in the mutated group median OS was 19 months (CI95% 15–23): patients treated with chemotherapy plus target therapy reached a median OS of 27 months (CI95% 15–40) compared with patients treated with chemotherapy alone for which the median OS was 14 months (CI95% 9–19; p = 0.02).

These results were confirmed in the ≥80 years old patients (data not shown).

In KRAS wild type group median OS was 25months (CI95% 20–30); median OS in patients treated with chemotherapy plus target therapy was 25 months (CI95% 16–34) compared with patients treated with chemotherapy alone for those median OS was 29 months CI95% 18–39; p = 0.37).

No difference in results was noted in the ≥80 years old patients (data not shown).

In 48 patients (32%) was administered cetuximab and in 34 (23%) bevacizumab.

No statistically significant relationship about comorbidities and age was noted.

The analysis of prognostic factors for OS at multivariate analysis showed that the statistically significant variables are age, performance status, chemotherapy, surgery of metastasis, surgery of primary tumor (Table 2).

**Table 2:** Analysis of prognostic factors.

**Variables**	**Univariate Analysis**		**Multivariate Analysis**	
	**HR (CI 95%)**	*p-value*	**HR (CI 95%)**	*p-value*
Sex (M* vs F**)	1.24 (1.03–1.49)	0.02	1.21(1.01–1.46)	*0*.*04*
Age (≥80 vs <80)	1.74 (1.45–2.09)	<0.0001	1.75 (1.45–2.12)	*<0*.*0001*
ECOG PS (2 vs 0/1)	3.56 (2.82–4.48)	<0.0001	2.51 (1.94–3.25)	*<0*.*0001*
Synchronous met. *** (yes vs no)	1.16 (0.97–1.40)	0.11	-	*n*.*s*.
N. of site of met. (>1 vs 1)	1.23 (1.02–1.48)	0.03	-	*n*.*s*.
Site of met. (liver vs other)	1.27 (1.04–1.55)	0.02	1.33 (1.09–1.63)	*0*.*006*
Chemotherapy (no vs yes)	2.19 (1.77–2.72)	<0.0001	2.14 (1.68–2.73)	*<0*.*0001*
Surgery of met. (no vs yes)	2.55 (1.95–3.33)	<0.0001	2.48 (1.88–3.29)	*<0*.*0001*
Surgery of primary tumor (no vs yes)	2.14 (1.71–2.68)	<0.0001	1.66 (1.31–2.11)	*<0*.*0001*
Site of primary tumor (colon vs rectum)	1.04 (0.85–1.27)	0.74	-	*n*.*s*.
Comorbidity (yes vs no)	1.01 (0.83–1.23)	0.92	-	*n*.*s*.

*M: male

**F: female

*** met: metastasis

**Fig 4:**
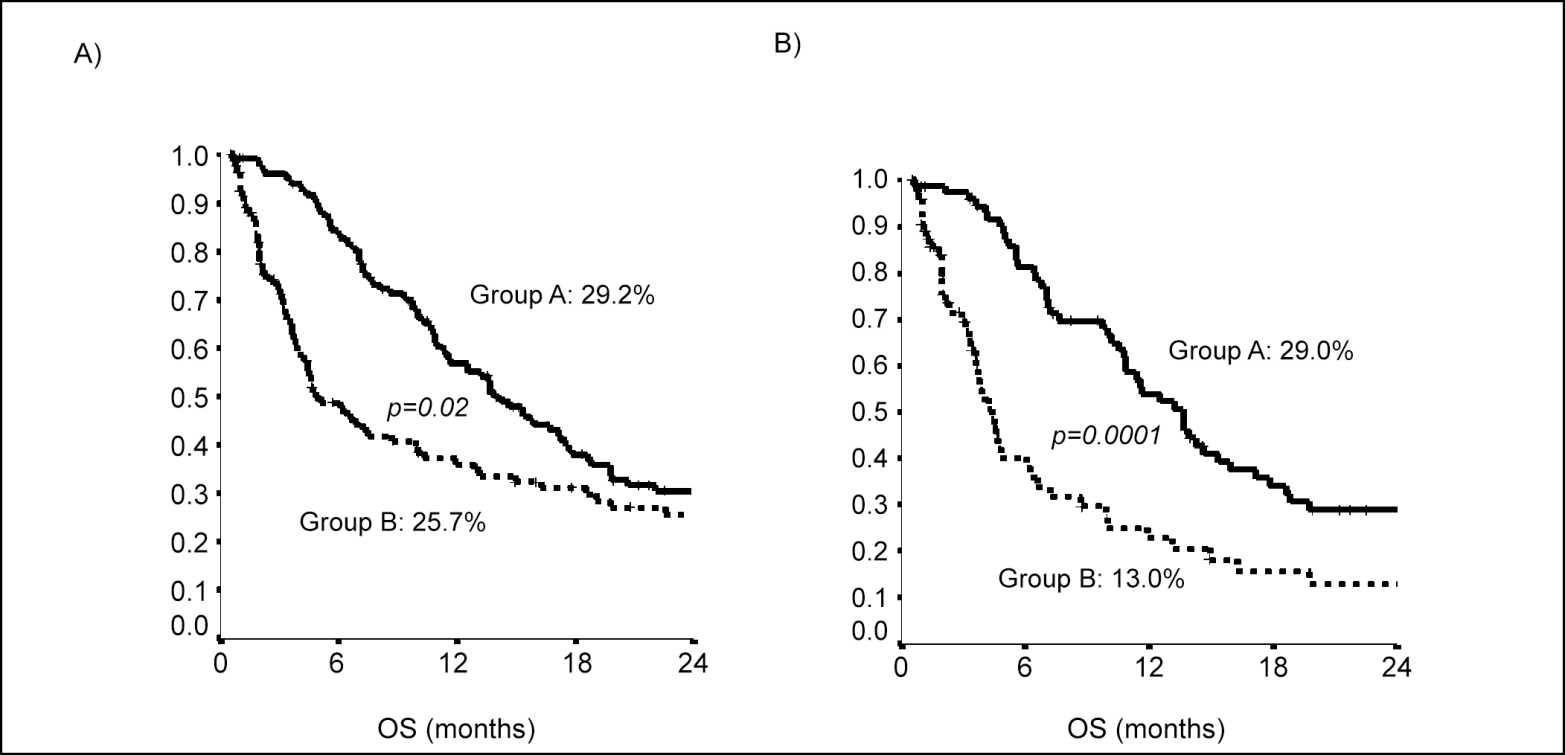
Overall Survival curves adjusted for propensity score: a) patients treated with chemotherapy, b) patients treated with chemotherapy over 80 years.

The use of propensity score used to reduce bias of retrospective observational study showed that the group A and B were homogeneous for performance status, surgery and number of patients; the OS curves adjusted for propensity score were statistical significant also in the group of patients over 80 years Fig 4.

## Discussion

Elderly patients with metastatic disease receive treatment (surgery, chemotherapy or radiotherapy) less often than younger patients and many of them do not receive what is considered as the standard approach. Consequently, limited data are available on the risks and benefits of specific anticancer treatments in elderly patients [23].

The first study that showed data about toxicities and efficacy of first line chemotherapy regimen in elderly patients was published in 2013. It showed a significant improving in progression-free survival with the association of an oral fluoropyrimidine (capecitabine) and an anti-VEGF (bevacizumab) with a safety profile [14].

The retrospective analysis of our data indicated that elderly patients, included those with age >80 years old or with a poor performance status could benefit from treatment (respectively p<0.0001 and p<0.0001).

Moreover, surgery is a valid treatment for this group, also when used for liver metastasis.

In the present study the median overall survival of patients treated with chemotherapy was 20 months. This is an interesting result if we consider that the data in the available literature showed a median overall survival in the general population that reached about 30 months [24–26].

The KRAS analysis was performed in only 35% of the patients. We observed that in the mutated group the addition of target therapy improved OS that reached 27 months (p = 0.02) (data confirmed also in patients with ≥ 80 years), while it seemed to be not confirmed in the KRAS wild type group (p = 0.37).

The small number of patients did not allow to make a definitive conclusion about it.

It is well known that some drugs, including the cytotoxics used in the treatment of cancer, have different metabolism and toxicity in elderly patients compared to younger patients [27–28] but in our study we noted that only 12.5% of patients discontinued treatment for toxicities. Furthermore, 21% of patients that started chemotherapy underwent third line treatment. The difference between chronologic and biologic age is a key point to evaluate. Formal geriatric and comorbidity assessments were not done for all patients because at the start of the study they were not widely used.

The routine use of validated tool, which include functional, mental, and clinical status, represent an essential support to select better patients who could benefit from heavy treatments and to find the optimal treatment to increase survival for therapeutic trials focused on elderly patients.

Moreover, older patients with adequate performance status and functional status and reasonable life expectancy should receive surgery and multi-agent chemotherapy.

In conclusion, the results of the present study show that in clinical practice treatment has a positive effect on elderly patients overall survival confirmed at multivariate analysis. It is reasonable to offer them the standard of care used for the treatment of younger patients with a careful selection that includes also the KRAS status although it deserves further evaluation because of the small number of patients analyzed.

Older patients with good PS and without comorbidities that might increase their risk of treatment-related toxicities should be considered for combination chemotherapy, possibly in association with a targeted agent. On the other hand, to less-fit patients it should be offered a monochemotherapy alone: our study results evidenced also an increasing of OS in this treated subgroup with a less risk of toxicities. When any treatment is feasible, patients might undergo to best supportive care alone keeping in mind that a good treatment of symptoms improves survival [29].

A greater participation of elderly patients in clinical trials is essential.
